# Successful Rotational Atherectomy for an Angulated Calcified Lesion in an Anomalous Right Coronary Artery Using the “Mother-and-Child” Technique

**DOI:** 10.1155/2018/5927161

**Published:** 2018-01-14

**Authors:** Manabu Ogita, Satoru Suwa, Taketo Sonoda, Shuta Tsuboi, Katsumi Miyauchi, Hiroyuki Daida

**Affiliations:** ^1^Department of Cardiology, Juntendo University Shizuoka Hospital, Izunokuni, Japan; ^2^Department of Cardiovascular Medicine, Juntendo University Graduate School of Medicine, Tokyo, Japan

## Abstract

Percutaneous coronary intervention (PCI) involving the anomalous coronary artery is challenging with respect to difficulty in achieving stable catheterization. Rotational atherectomy (RA) can facilitate severely calcified lesions to improve stent delivery and stent expansion; however, its utility in tortuous and angulated coronary arteries is limited with difficulty in delivery of the RA burr. The mother-and-child technique is effective for complex PCIs with increased backup force for device delivery in such complicated cases. We report a case of successful rotational atherectomy using the “mother-and-child” technique with a Dio thrombus aspiration catheter for an angulated calcified lesion in an anomalous origin of the right coronary artery.

## 1. Introduction

Coronary artery anomalies are a diverse group of congenital disorders that are found in 5% of the population [1]. Anomalous origin of the right coronary artery (RCA) accounts for 10% of all anomalous coronary artery cases [2]. Percutaneous coronary intervention (PCI) involving the anomalous coronary artery is challenging with respect to selection of the guide catheter, insufficient backup force, and device delivery. The mother-and-child technique is useful for improving backup support and delivery of stents in such lesions [3]. Rotational atherectomy (RA) can facilitate lesion and stent expansion in severely calcified lesions especially in patients undergoing hemodialysis [4]; however, its utility is limited in tortuous and angulated coronary arteries, with an increased risk of vessel perforation and difficulty in delivery of the RA burr. We describe a case of successful rotational atherectomy for an angulated calcified lesion in the anomalous right coronary artery using the “mother-and-child” technique.

## 2. Case Presentation

A 46-year-old man with end-stage renal disease due to diabetic nephropathy was referred to our institution for percutaneous coronary intervention (PCI) for a severe calcified lesion in an anomalous right coronary artery. He had been undergoing hemodialysis three times a week for 3 years. Since patients with diabetic ESRD are at a high risk for coronary artery disease, coronary computed tomography angiography (CTA) had been performed at his previous hospital visit, which revealed multivessel disease. Coronary angiography (CAG) showed severe stenosis in the midportion of the left anterior descending (LAD) artery and a chronic total occlusion (CTO) in the proximal portion of the right coronary artery with an anomalous origin. Hence, PCI of the LAD lesion was first performed, and a bioresorbable polymer sirolimus-eluting stent (Ultimaster 3.0 × 38 mm and 3.0 × 33 mm, TERUMO, Tokyo, Japan) was successfully implanted at the previous hospital. Staged PCI for CTO of the RCA was performed with successful wire crossing and recanalization. However, since subsequent standard balloon angioplasty was unsuccessful, he was referred to our hospital for PCI of the anomalous RCA.

**Figure 1:**
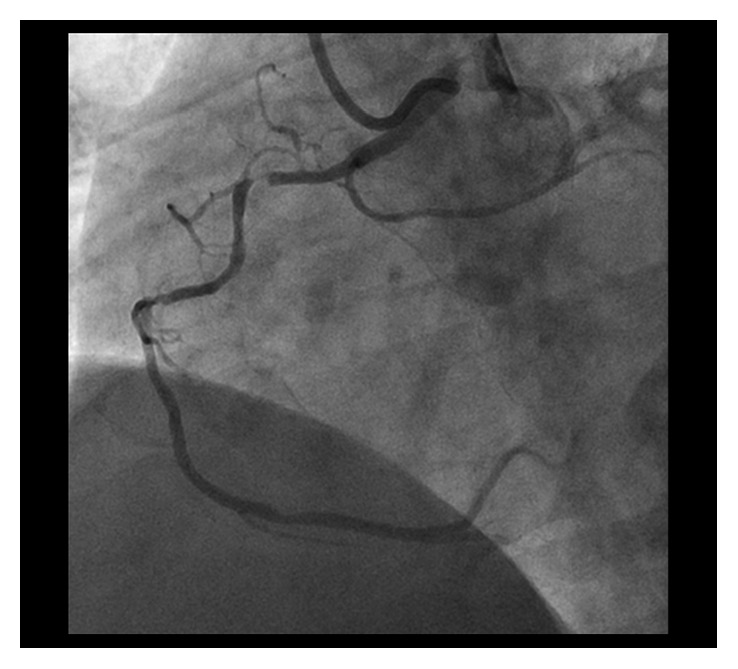
The left anterior oblique coronary angiography view showed severe calcified stenosis of the proximal segment of the right coronary artery (RCA) with anomalous origin.

**Figure 2:**
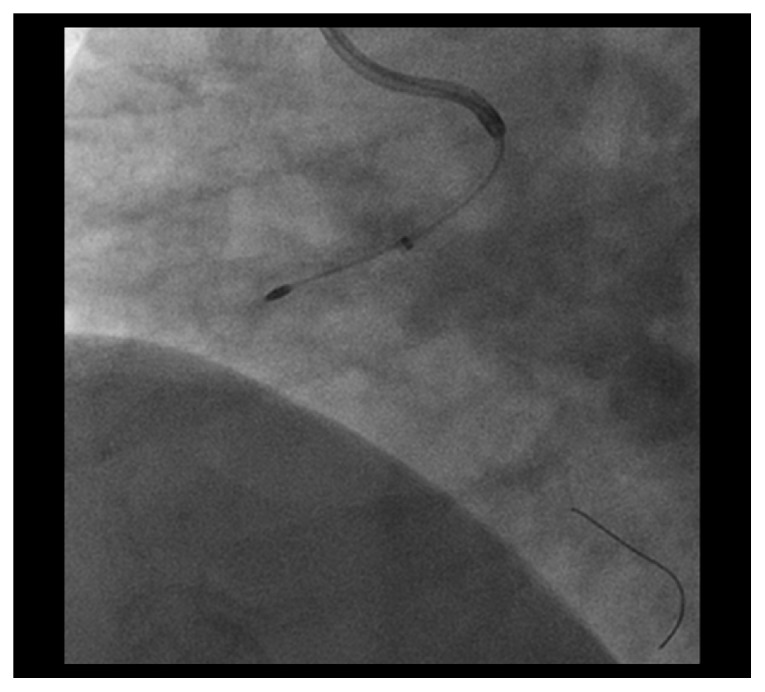
Advancement of a 1.5 mm burr through the Dio thrombus aspiration catheter.

**Figure 3:**
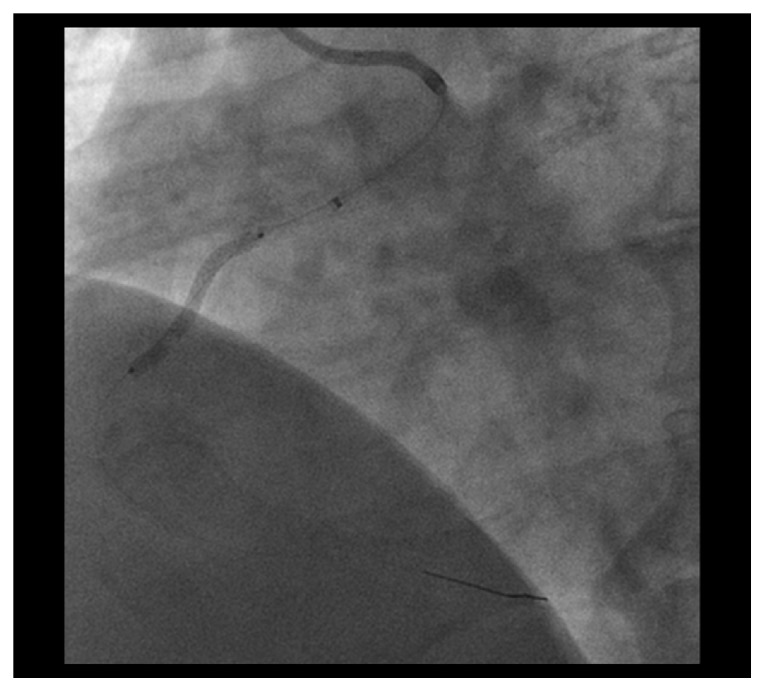
Successful stent deployment within the lesion.

**Figure 4:**
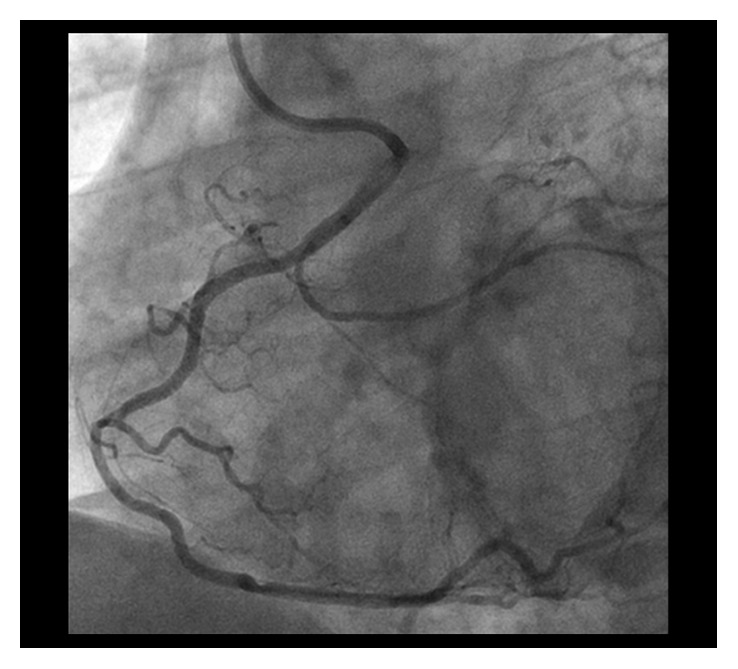
Final angiographic results after stent implantation and after dilatation.

CAG demonstrated residual severely calcified stenosis in the proximal RCA with patent antegrade coronary flow (Figure 1). We selected right femoral access with a long sheath using a 7 Fr Amplatz left 2 guiding catheter (Asahi Intecc, Tokyo, Japan) in consideration of size up to a 2.0 mm burr. A SION blue wire (Asahi Intecc) was advanced into the distal RCA using a microcatheter (Caravel, Asahi Intecc) and intravascular ultrasonography (IVUS; OptiCross, Boston Scientific Corp., MA, USA). Based on the IVUS findings, which indicated severe circumferential calcification with a minimal luminal diameter larger than 1.25 mm, we decided to perform rotational atherectomy with a 1.5 mm burr (Boston Scientific Corp., MA, USA). For this, the first wire was exchanged for an extrasupport RotaWire (Boston Scientific) using the microcatheter. To obtain greater backup force, a Dio thrombus aspiration catheter (Goodman Co. Ltd., Aichi, Japan) was inserted into the RCA. The burr was successfully delivered to the anomalous RCA with dynaglide because we had difficulty advancing the burr manually. Halfway ablation with a 1.5 mm burr at 200,000 rpm was attempted several times, taking care not to advance the burr beyond the angle [5], although subsequent CAG and IVUS did not show the effective lesion modification after ablation. Then, we switched to balloon angioplasty with a 2.5 mm high-pressure balloon (NC Emerge 2.5 × 12 mm, Boston Scientific) and a 2.5 mm cutting balloon (Flextome 2.5 × 10 mm, Boston Scientific), but it was not enough to dilate the lesion. Finally, we switched back to rotational atherectomy and were subsequently able to pass the burr beyond the angulated calcified lesion without complications (Figure 2) and confirmed a crack using IVUS. Following effective dilation with a 3.0 mm high-pressure balloon (CELUSUS 3.0 × 8 mm, NIPRO, Osaka, Japan), we deployed a bioresorbable polymer everolimus-eluting stent (Synergy 2.5 × 24 mm, Boston Scientific) (Figure 3). The stent was additionally dilated with a noncompliance balloon (CELUSUS 3.0 × 8 mm, NIPRO, Osaka, Japan). A final coronary angiogram showed adequate and favorable dilatation of the culprit lesion (Figure 4).

## 3. Discussion

We report a case of successful rotational atherectomy for an angulated calcified lesion in an anomalous right coronary artery using the “mother-and-child” technique. PCI for the anomalous RCA can be associated with difficulty in achieving stable catheterization, leading to reduced backup support and device delivery failure. Rotational atherectomy (RA) can facilitate lesion and stent expansion in severely calcified lesions, although its utility is limited in tortuous and angulated coronary arteries, with an increased risk of complications. Appropriate guiding catheter selection is a key step to ensure successful PCI in such cases.

The mother-and-child technique is effective for complex PCIs, as it increases the backup force through deep catheterization of the target vessel and facilitates device delivery in tortuous or calcified lesions. Several mother-and-child catheters have been introduced in clinical practice (Table 1). The GuideLiner catheter is a guide extension catheter with the monorail system which permits rapid exchange and now is, hence, increasingly used [6]. Only a few cases have been previously reported of use of the mother-and-child technique to deliver rotational atherectomy burrs. Vo et al. first reported the use of the GuideLiner catheter to deliver rotational atherectomy burrs in tortuous vessels [7]. Costanzo et al. also reported GuideLiner-facilitated rotational atherectomy in calcified right coronary arteries [8]. However, the mother-and-child technique for rotablation has not been well established. To the best of our knowledge, this is the first report of RA using the mother-and-child technique in an RCA with anomalous origin.

In our case, we selected the conventional mother-child catheter, the 5 Fr Dio aspiration catheter for the following reasons. First, the 5 Fr Dio thrombus aspiration catheter has the RX-type inner catheter which facilitates smooth delivery and deep insertion of the outer catheter into the anomalous RCA. The relatively large inner lumen size of the outer catheter (1.51 mm) can provide the strong backup force for device delivery. Second, there is a concern that the rapid exchange transition zone of GuideLiner can lead to less backup support, difficulty in delivering the burr, and increase in the risk of burr entrapment. Thus, we thought that the Dio thrombus aspiration catheter can provide stronger backup support in our patient with rather complicated anatomy. Komatsu et al. reported successful PCI in an anomalous RCA using the Dio thrombus aspiration catheter [9]. This catheter allows not only stent delivery but also rotating burr delivery to severe calcified lesions in anomalous RCAs and can be a useful adjunctive device for rotational atherectomy. It is essential for operators to position the burr distal to the Dio thrombus aspiration catheter to prevent potential damage and shear of the child catheter. In summary, the “mother-and-child” technique is useful for rotational atherectomy in anomalous angulated RCAs. Rotablation with a Dio thrombus aspiration catheter is a potential therapeutic strategy in such complicated cases.

## 4. Conclusion

We report a case of successful rotational atherectomy using the “mother-and-child” technique with a Dio thrombus aspiration catheter for an angulated calcified lesion in an anomalous origin of the right coronary artery.

## Figures and Tables

**Table 1:** Design comparison of the guide extension catheter available in Japan and Dio thrombus aspiration catheter.

	Guidezilla II (Boston Scientific)	GuideLiner V3 (Lifeline)	Guideplus (NIPRO)	Dio (Goodman)
Sizes	6 Fr, 7 Fr, 8 Fr, and 6 Fr long	5.5 Fr, 6 Fr, 7 Fr	6 Fr	5 Fr
Inner lumen		1.30 mm (5.5 Fr)	1.30 mm (6 Fr)	1.51 mm (5 Fr)
	1.45 mm (6 Fr)	1.42 mm (6 Fr)		
	1.60 mm (7 Fr)	1.57 mm (7 Fr)		
	1.83 mm (8 Fr)	1.80 mm (8 Fr)		
Outer lumen		1.60 mm (5.5 Fr)	1.50 mm (6 Fr)	1.72 mm (5 Fr)
	1.45 mm (6 Fr)	1.70 mm (6 Fr)		
	1.60 mm (7 Fr)	1.90 mm (7 Fr)		
	2.11 mm (8 Fr)	2.16 mm (8 Fr)		
Guide segment length	25 cm/40 cm (6 Fr long)	25 cm	25 cm	
Working length	150 cm	150 cm	145 cm	124 cm
Coating	Z-glide (hydrophilic)	Silicon wipe (hydrophilic)	Hydrophilic	Hydrophilic
